# Optimization of a Digital Mass Filter for the Isolation of Intact Protein Complexes in Stability Zone 1,1

**DOI:** 10.1021/acs.analchem.2c05221

**Published:** 2023-01-26

**Authors:** Robert L. Schrader, Thomas E. Walker, Sumeet Chakravorty, Gordon A. Anderson, Peter T. A. Reilly, David H. Russell

**Affiliations:** † Department of Chemistry, Texas A&M University, College Station, Texas77843, United States; ‡ Department of Chemistry, Washington State University, Pullman, Washington99164, United States; § GAA Custom Engineering, Kennewick, Washington99338, United States

## Abstract

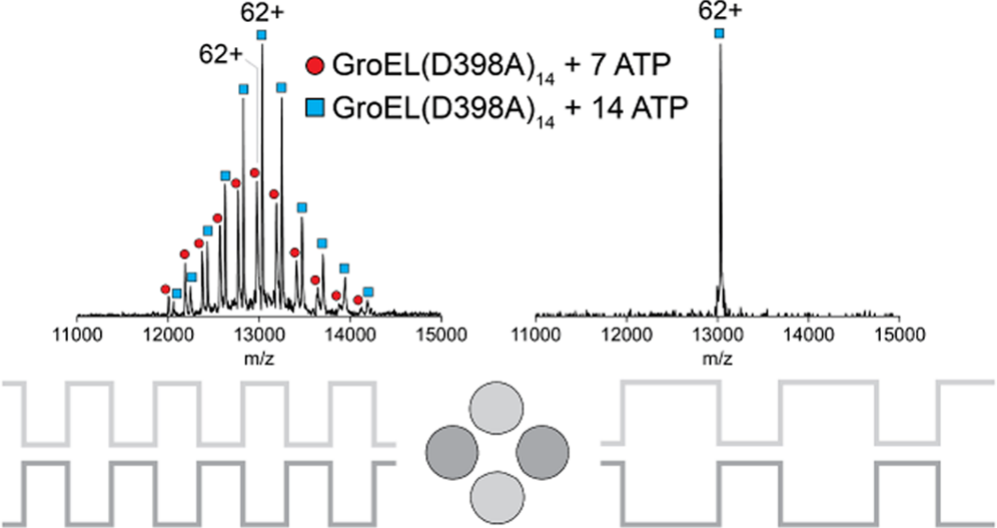

Digital mass filters are advantageous for the analysis of large molecules due to the ability to perform ion isolation of high-*m*/*z* ions without the generation of very high radio frequency (RF) and DC voltages. Experimentally determined Mathieu stability diagrams of stability zone 1,1 for capacitively coupled digital waveforms show a voltage offset between the quadrupole rod pairs is introduced by the capacitors which is dependent on the voltage magnitude of the waveform and the duty cycle. This changes the ion’s *a* value from *a* = 0 to *a* < 0. These effects are illustrated for isolation for single-charge states for various protein complexes up to 800 kDa (GroEL) for stability zone 1,1. Isolation resolving power (*m*/Δ*m*) of approximately 280 was achieved for an ion of *m*/*z* 12,315 (*z* = 65+ for 800.5 kDa GroEL D398A), which corresponds to an *m*/*z* window of 44.

## Introduction

Electrospray ionization of large (100 kDa to MDa) biomolecules produces a distribution of multiply charged ions, and each of the charge states is further broadened by their isotopic distribution as well as salts and adducts.^1−3^ These broader peak widths impose limitations for many types of mass spectrometry-based biochemical and biophysical studies, especially studies of protein–metal,^4^ protein–lipid,^5^ protein–protein,^6^ and protein–nucleotide^7^ interactions. These limitations may in some cases be overcome by combining charge-reducing reagents to provide greater separation between charge states. Charge-reducing reagents can also alter and/or compete with the formation of desired complex or reduce the stabilities of the complexes.^7−9^ Such peak broadening compromises our capabilities to mass-select individual charge states for structural analysis using tandem mass spectrometry (MS) (*vis*. collision-induced dissociation (CID),^10^ surface-induced dissociation (SID),^11^ electron capture dissociation (ECD),^12,13^ ultraviolet photodissociation (UVPD),^14^ and/or ion mobility-MS^15^). Quadrupole mass filters coupled with Orbitraps^16−18^ or time-of-flight mass analyzers^19,20^ are commonly used for tandem MS using quadrupoles with reduced radio frequency (RF) drive frequencies, reducing the necessary voltage output to achieve the desired mass range. A digitally driven quadrupole circumvents this issue by adjusting the RF drive frequency instead of the voltage.

The stability of ions in the quadrupole mass filter, driven digitally or sinusoidally, are defined in terms of Mathieu parameters *q* and *a*
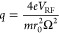
1
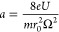
2where *e* is the elementary charge, *V*_RF_ is the zero to peak RF voltage, *m* is the mass of the ion, *r*_0_ is the rod radius, Ω is the angular frequency of the RF, and *U* is the 0 to rod DC voltage applied between the rod pairs.^21,22^ Stability diagrams define specific values of *a* and *q* where an ion will be stable in both the *x* and *y* dimension.^23^ For the digital quadrupole, the duty cycle of the waveform also affects the stability diagram.^24^ The duty cycle defines the percentage of the waveform at value *V*_RF_ with the remainder at −*V*_RF_. This means for a 61.2/38.8 duty cycle, the waveform is *V*_RF_ for 61.2% of the waveform period and −*V*_RF_ for 38.8% of the waveform period. As with a sinusoidal quadrupole, identical waveforms are applied to the rod pairs 180° out of phase. The 50.0/50.0 duty cycle will act as an ion guide and allow all ions greater than the low mass cutoff to be transmitted. This is analogous to a sinusoidal RF quadrupole operating with no DC voltage applied between the rod pairs.

Traditionally, sinusoidal waveforms generated by a resonant circuit are applied to opposite rod pairs of precisely machined hyperbolic rods. Ions are scanned through the apex of the stability diagram at *q* = 0.706, *a* = 0.23699 by adjusting the RF voltage amplitude and the DC voltage between the rod pairs.^22^ For large *m*/*z* ions, this voltage becomes very large and prohibitive. Multiple methods have been developed to reduce the necessary voltage outputs such as reducing the RF drive frequency of the quadrupole^16,18,25−27^ and scanning the RF drive frequency with a constant voltage.^28,29^

The sinusoidal quadrupole ion trap offers unique ways of increasing the upper mass limit compared to the quadrupole mass filter without increasing the voltage requirements. Resonance ejection is typically performed with a supplementary frequency near the low mass cutoff of the ion trap, but by reducing this frequency the mass range can be increased without increasing the voltage requirement.^30,31^ This supplementary frequency can also be scanned through the secular frequencies of the ions in the trap with a constant RF voltage to generate a mass spectrum with an increased mass range.^32,33^ Alternatively, ions can be ejected by applying a DC voltage until they reach the β = 0 stability boundary.^34,35^

The digital quadrupole mass filter (DigiQ) offers an alternative to the traditional sinusoidal mass filter.^36,37^ Unlike traditional sinusoidal mass filters where the RF voltage is adjusted, digital mass filters adjust the drive frequency and duty cycle maintaining a constant RF voltage to change stability conditions. Using digital waveforms, the stability diagram can be manipulated by changing the duty cycle to move stability zones to *a* = 0.^24,38^ This removes the requirement for scanning the DC voltage to perform ion isolation in a mass filter. By removing the necessity for generating very high voltages, high mass-to-charge ions can be readily detected.^39−43^

Coupling RF and DC signals through capacitors and resistors is necessary to create the axial DC gradient through the instrument. When used for coupling digital waveforms where the duty cycle is not always 50.0/50.0, this creates a voltage and duty cycle-dependent DC offset between the rod pairs. The effect of this offset is investigated here and the isolation performance for native protein complexes is illustrated for stability zone 1,1.

## Experimental Section

### Chemicals

Human recombinant C-reactive protein (CRP, 115 kDa, solution in tris-buffered saline, 2 mM CaCl_2_, 0.05% NaN_3_, pH 7.5) was purchased from Lee Biosolutions (Maryland Heights, MO). High-performance liquid chromatography (HPLC)-grade water was purchased from Millipore-Sigma (St. Louis, MO). Ammonium acetate (AA), ethylenediamine diacetate (EDDA), triethyl ammonium acetate (TEAA), and adenosine 5′-triphosphate disodium hydrate salt (ATP) were purchased from Sigma-Aldrich (St. Louis, MO). GroEL D398A mutant (800 kDa) was expressed and purified as previously described.^44^ CRP was buffer-exchanged into aqueous 200 mM ammonium acetate solutions using a Bio-Rad (Hercules, CA) Micro Bio-Spin P6 Column. GroEL was buffer-exchanged into aqueous 200 mM ammonium acetate, 200 mM ethylenediamine diacetate, or 160 mM ammonium acetate/40 mM triethyl ammonium acetate using the same column. Solutions were adjusted to working concentrations of 1–5 μM. For GroEL-ATP experiments, aqueous ATP was added to the GroEL solution to a final concentration of 500 nM GroEL D398A and 2.5 μM ATP in 200 mM ammonium acetate.

Borosilicate capillaries (10 cm length, 1.5 cm o.d., 0.86 cm i.d.) were purchased from Sutter Instruments (Navajo, CA) and pulled to a 1–5 μm tip using a P100 tip puller (Sutter Instruments). Protein samples were loaded into the capillary and high voltage (1.1–1.5 kV) was applied through a platinum wire.

### Instrumentation

The dual quadrupole instrument used here has been described previously.^41^ In this study, the ion mobility drift tube was removed and some modifications were made to the source region. A rendering of the instrument is provided in Figure S1. Briefly, ions enter an RF ion funnel (1.5 Torr, 470 kHz, 250 V_p–p_) from a heated capillary maintained at 120 °C. The ions then enter the second vacuum region, maintained at approximately 300 mTorr, that contains a short 3.5 mm *r*_0_ square quadrupole (*q0*, 1.3 MHz, 300–500 V_p–p_). To activate the ions to remove adducted species, a voltage drop is imposed between the ion funnel and *q0*. The ions were trapped (4 ms) and ejected (1.5 ms) by modulating the DC voltage of *q0* and the quadrupole entrance lens, both by DEI PVX-4145 Pulsers (Directed Energy, Inc., Fort Collins, CO). This process is performed similarly to in-source trapping that has been implemented on the Thermo Fisher Q Exactive UHMR Orbitrap.^18^ In the third vacuum region (maintained at 8 × 10^–4^ Torr), the ions travel through a 4 mm *r*_0_ hyperbolic quadrupole (Thermo Fisher, Part Number 80100-60109) of 250 mm length (1.4 MHz, 250–900 V_p–p_) and a 4.75 mm *r*_0_ octopole (750 kHz, 200 V_p–p_). In the fourth vacuum region (maintained at 10^–5^ Torr) the ions travel through an identical 4 mm *r*_0_ hyperbolic quadrupole operated with digital waveforms and a 3.5 mm *r*_0_ octopole (850 kHz, 200 V_p–p_). Following a skimmer, the ions enter a fifth vacuum region (10^–5^ Torr) with a 4.75 mm *r*_0_ octopole (900 kHz, 200 V_p–p_) and enter the HCD cell of the Thermo Fisher Exactive Plus EMR Orbitrap mass spectrometer (Bremen, Germany), as described previously.^45^ DC voltages are applied using a Modular Intelligent Power Supply (MIPS) System power supply (GAA Custom Engineering, Kennewick, WA). RF is supplied using MIPS High Q RF heads from GAA Custom Engineering.

The second quadrupole is used for mass selection using digital waveforms that are synthesized using a low-voltage waveform generator (LV-WFG). The high-voltage waveforms are generated by two DEI PXI-4145 pulsers. The LV-WFG utilizes the comparison method of rectangular waveform generation in which direct digital synthesis (DDS) is used to create a stepped waveform. The stepped waveform is smoothed via a low-pass filter to remove the steps and create waveforms that have frequency resolution between 32- and 48-bit. The LV-WFG houses a field-programmable gate array (FPGA), low-pass filter, analog comparators, digital-to-analog convertor (DACs), and waveform shaping electronics. The programmed FPGA can rapidly and precisely create or switch any periodic stepped waveform via the DDS method. The DDS creates triangular waves that have the inherent characteristic of constant slopes over most of the wave. Here, the triangular waves are used to generate rectangular waves that are created by comparison to nonzero potentials that are created with the DACs. The comparator switches its output to the high state when the triangular voltage is greater than the comparator voltage and switches to the low state when it is not. The inherent characteristic of triangular waves has an advantage over sine waves during the comparator generation process because the duty cycle resolution does not change at extreme duty cycles so that the resolution at 90.0/10.0 is the same as a 50.0/50.0 duty cycle. Power for the waveforms is applied using an XP Power model PLS6004002.5 (Pangbourne, U.K.). A voltage of 150 V_0–p_ was used with frequencies varying from 400 to 75 kHz. The digital waveform was applied to the rods in two different configurations: (1) directly from the DEI pulser with the DC potential bias applied by offsetting the input voltages and (2) by capacitively coupling the waveform to the rods using a custom offset box developed by GAA Custom Engineering.

## Results and Discussion

**Figure 1:**
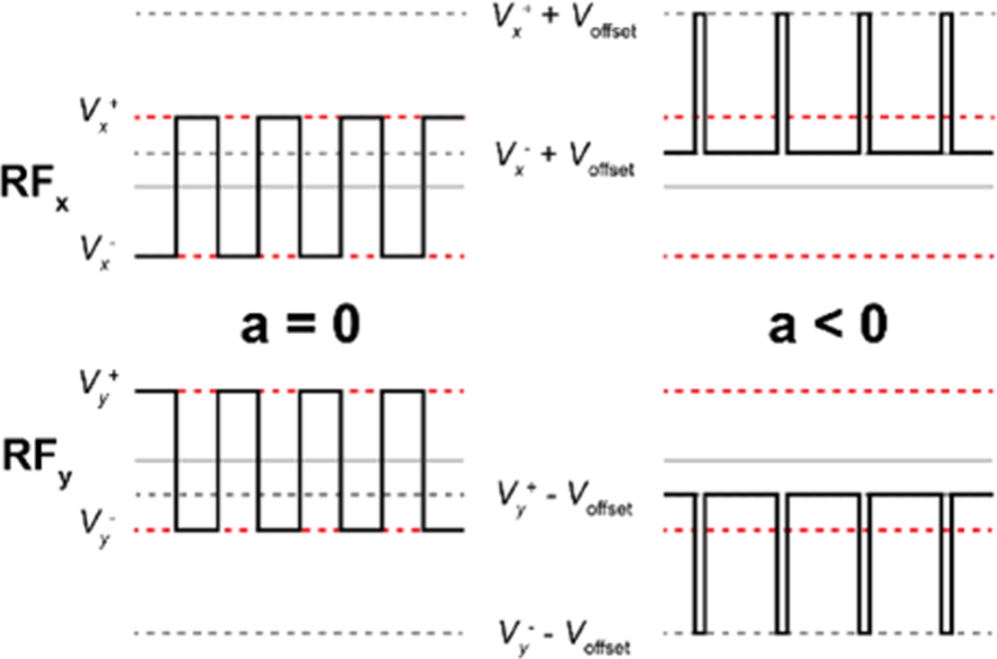
Waveforms applied to the quadrupole rod pairs have magnitude *V*^+^ and *V*^–^ and at a 50.0/50.0 waveform are centered around the DC bias value. As the duty cycle is adjusted for ion isolation, the waveform is adjusted by an offset voltage (*V*_offset_) which makes the time-averaged voltage across the capacitor zeros. The magnitude of *V*_offset_ is dependent on the magnitude of *V*^+^ and *V*^–^ and the duty cycle.

Capacitive coupling of the digital waveform to the quadrupole rods was necessary to apply a DC bias necessary to generate the axial DC gradient through the instrument. Unlike sinusoidally driven quadrupoles, capacitive coupling creates a problem for digitally driven quadrupoles. Because the time-averaged voltage across the capacitor must be zero, the value of voltage applied to the quadrupole (*V*^+^ and *V*^–^) will only be *V*^+^ and *V*^–^ for a 50.0/50.0 duty cycle. At any other duty cycle, the time-averaged voltage of the waveform is nonzero. Consequently, the voltage of the waveforms will be offset by the value necessary for the time-averaged voltage of the waveform to be zero. Furthermore, the value of *V*^+^ and *V*^–^ will be reduced for the higher-duty-cycle waveform and will be larger for the lower-duty-cycle waveform as shown in Figure 1. This results in the ions having a negative value of *a* that must be corrected by the applied DC voltages to perform ion isolation at *a* = 0 as desired. As the voltage offset is the result of the capacitors, the magnitude of this value is a function of the magnitude of the RF voltage and the duty cycle.

**Figure 2:**
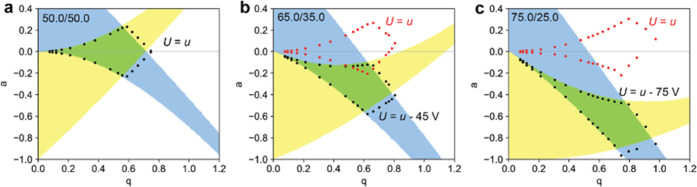
Mathieu stability diagram calculated from matrix methods superimposed with experimentally calculated boundaries for (a) 50.0/50.0 duty cycle, (b) 65.0/35.0 duty cycle, and (c) 75.0/25.0 duty cycle. Red points are calculated with uncorrected *U* values, which are affected by the capacitive coupling whereas black points incorporate the corrected voltage.

The Mathieu stability diagram for stability zone 1,1 was determined experimentally for a 50.0/50.0 duty cycle (Figure 2a) by determining the stability of the 24+ charge state of CRP for voltage and frequency values near the boundary. The boundary was determined by finding the parameters for which the ion was removed from the Orbitrap mass spectrum and thereby unstable in the quadrupole mass filter. Experimental points are plotted over the theoretical stability diagram^24^ (Figure 2). The stability diagram for the 50.0/50.0 duty cycle is most like that of a sinusoidal mass filter, where the *q* axis is scaled by a factor of 4/π.^46^ In the case of the 50.0/50.0 duty cycle, the value of *a* could be directly determined from the voltage offset (Figure 2a). We will refer to the voltage offset as *u*, where *u* is the 0 to rod DC voltage applied between the rod pairs. In the case of the 50.0/50.0 waveform, *U* = *u*, which is unique to this duty cycle, as the time-averaged voltage across the capacitor is zero. For all other duty cycles, the value of *U* must be corrected to account for the voltage offset due to the capacitors.

A similar method was employed to plot the stability diagram for zone 1,1 at a 65.0/35.0 duty cycle (Figure 2b). Unlike the 50.0/50.0 duty cycle, determining *a* directly from the difference between the DC voltage applied to the rods (*u*, plotted in red) does not give the correct stability diagram. The correct value of *U* can be calculated from the waveform parameters and the applied DC voltages. For the 65.0/35.0 duty cycle and 150 V_0–p_, the value of *U* is *U* = *u* – 45 V. The correction, 45 V in this case, is determined from the time-averaged voltage of the waveform for a given voltage and duty cycle. After determining *a* from the corrected values, the experimental points (plotted in black) match the theoretical stability diagram. This correction increases to 75 V for the 75.0/25.0 duty cycle (Figure 2c) as this duty cycle increases the time-averaged voltage.

Other authors have employed an alternative equation for *U* for digital quadrupoles rather than the definition used here.^47^ The equation by Ding et al. was not used here as it is only an approximation for stability zone 1,1 and is not complete or exact.^24^ While this equation does calculate the time-averaged voltage, this equation does not yield points that match the theoretical stability diagram (Figure S2).

**Figure 3:**
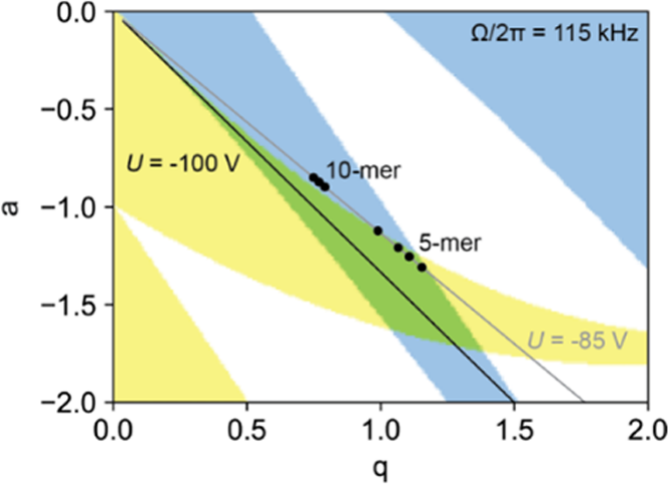
Mathieu stability diagram for a duty cycle of 0.85/0.15. The black line represents ions for a drive frequency of 115 kHz with *U* = −100 V, which is a result of the voltage offset between the rods induced by the capacitive coupling. Adjusting *U* to −85 V moves the ions to the tip of the stability diagram where isolation occurs in zone 1,1 at this duty cycle. Black dots represent the various charge states of CRP for this set of conditions showing the ability to isolate a single-charge state.

Previously, an 85.0/15.0 duty cycle and ∼15 V offset was used to isolate single-charge states of protein complexes.^41^ It was hypothesized that this isolation was performed in zone 3,1 which has a *q* value of 4.5 and an *a* value close to 0. To isolate ions, the DC voltage applied to the rods was adjusted until only single-charge states were transmitted. The work here shows that this isolation was not in zone 3,1 but in zone 1,1 that had been manipulated by the duty cycle such that the apex was at a value of *q* = 1.32 and *a* = −1.24. Based on the data acquired here, a duty cycle of 85.0/15.0 would result in *U* = −100 V. At a drive frequency of 115 kHz, the ions would fall on the black line shown in Figure 3. Adjusting the DC voltages such that *U* = −85 V, the ions fall on the gray line, which intercepts the apex of the stability diagram. The black dots represent the various charge states of the CRP 5-mer and 10-mer. The voltage offset caused by the capacitive coupling created a very large voltage offset between the rods that must be corrected to be at *a* = 0 as was desired.

**Figure 4:**
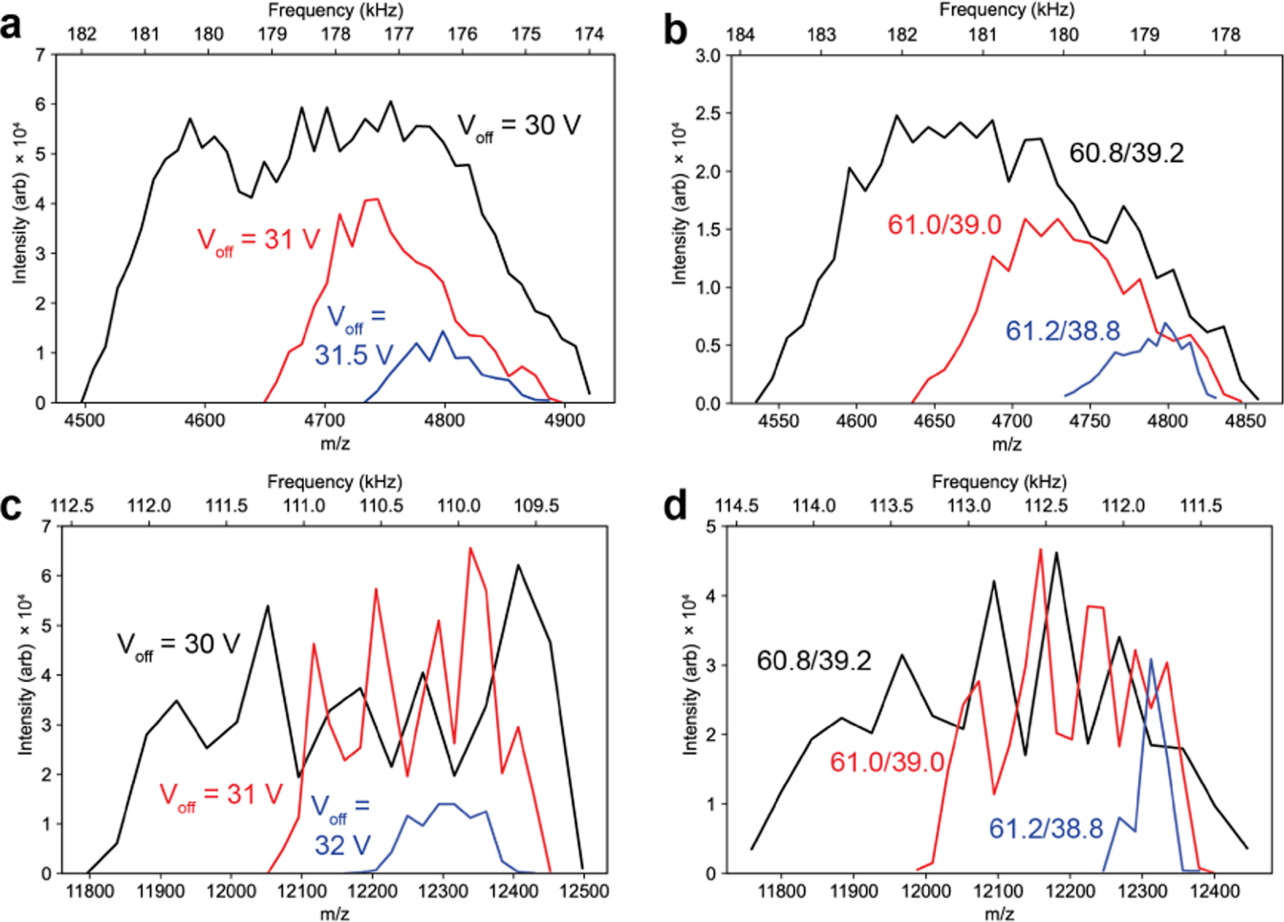
Ion intensity at *m*/*z* 4798 (24+ charge state of CRP) as a function of quadrupole drive frequency for (a) various values of *V*_offset_ with a constant duty cycle of 61.2/38.8 with the waveform capacitively coupled and (b) various duty cycles with the waveform applied directly. Ion intensity at *m*/*z* 12,310 (65+ charge state of GroEL) as a function of quadrupole drive frequency for (c) various values of *V*_offset_ with a constant duty cycle of 61.2/38.8 with the waveform capacitively coupled and (d) various duty cycles with the waveform applied directly. The *x* axis is plotted as a function of *m*/*z* calculated from *q* = 0.59 for the quadrupole drive frequency.

**Figure 5:**
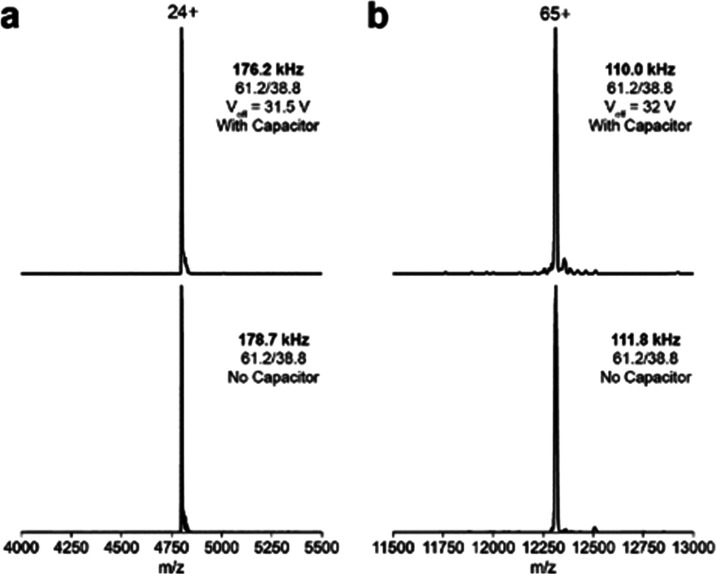
(a) Optimized mass spectrum for selection of the 24+ charge state of CRP with and without the waveform capacitively coupled. (b) Optimized mass spectrum for selection of the 65+ charge state of GroEL with and without the waveform capacitively coupled.

The resolving power achieved with a digital waveform isolation is dependent on the duty cycle as well as the value of *a*. The instrument was characterized in two modes: (1) with the waveforms and DC voltages capacitively coupled to the rods as previously described and (2) with the voltage pulser directly connected to the rods and adjusting the DC bias on the rods by changing *V*^+^ and *V*^–^. In a sinusoidally operated quadrupole, the resolution achieved is adjusted by changing the ratio of *a*/*q* near the apex of the stability diagram.^22^ Digitally operated quadrupoles operate similarly, but the stability diagram is manipulated by the duty cycle bringing the apex closer to *a* = 0 (Figure S3). For the capacitively coupled waveforms, a constant duty cycle of 61.2/38.8 was used, and the resolution was manipulated using the voltage offset. To calculate the resolution achieved by the quadrupole, the drive frequency was scanned, and the ion intensity was calculated from the Orbitrap ion intensity (Figure 4). For both CRP (Figure 4a) and GroEL (Figure 4c), the highest resolution is achieved for the voltage offset which moves the ions closest to *a* = 0, though the increased resolution comes at the expense of ion transmission. When the waveforms were applied directly to the rods, the duty cycle was used to manipulate the resolution as the ions are at *a* = 0. The same effect is observed for both CRP (Figure 4b) and GroEL (Figure 4d) where higher resolution is achieved when the ions are nearer to the apex at the expense of signal intensity. The highest resolving power achieved for GroEL is 279, which corresponds to a mass window at 12,310 of 44 *m*/*z*. At this resolution, the well depth of the ions is approximately 0.2 V, the same as a sinusoidal quadrupole operating with unit resolution.^48^ Higher operational voltages would increase the well depth (allowing for higher resolution), though this increases the power requirements for the high-voltage power supply and fringing fields. The mass spectrum observed for the highest resolution is shown in Figure 5. As expected, similar resolving power is achieved with and without capacitively coupling the waveforms. We hypothesize that the capacitor is loading down the voltage slightly. This can be observed in the approximately 2 kHz difference in the optimum ion selection frequency between the capacitively coupled selection and the direct selection.

**Figure 6:**
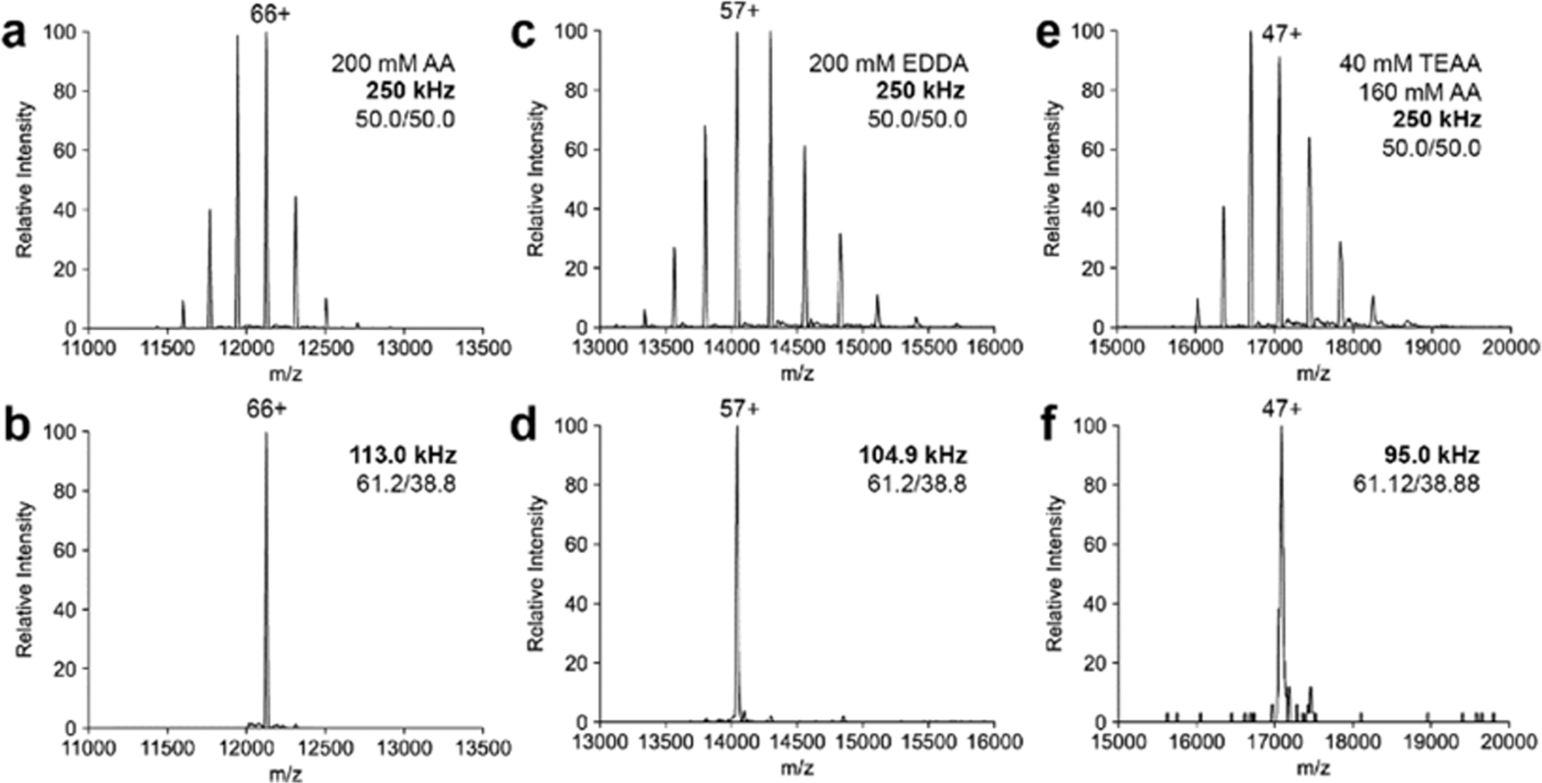
(a) Full-scan mass spectrum of GroEL in 200 mM ammonium acetate and (b) selection of the 66+ charge state with a 61.2/38.8 duty cycle. (c) Full-scan mass spectrum of GroEL in 200 mM ethylenediamine diacetate and (d) selection of the 57+ charge state with a 61.2/38.8 duty cycle. (e) Full-scan mass spectrum of GroEL in 160 mM ammonium acetate/40 mM triethyl ammonium acetate and (f) selection of the 47+ charge state with a 61.12/38.88 duty cycle.

For a sinusoidally operated quadrupole, the ratio of *a*/*q* must be increased to achieve a constant peak width. In other words, a linear scan of *a*/*q* will yield a constant resolution. A nonlinear scan of *a*/*q* yields a greatly preferred constant peak width.^22^ A scan of the frequency for a digitally operated quadrupole yields a constant resolving power (*q*/Δ*q*) as the *q* values are unchanged throughout the scan.^37^ Isolation at various *m*/*z* values was demonstrated by an isolation performed at three charge states: 66+, 57+, and 47+ (Figure 6). For both 66+ and 57+, a 61.2/38.8 duty cycle was used to achieve isolation of a single-charge state. In each case, the full-scan mass spectrum was acquired using a 50.0/50.0 duty cycle, 150 V_0–p_, and 250 kHz, which corresponds to a low mass cutoff of approximately *m*/*z* 2036. As the ions are trapped in the source region, the signal intensity is dependent on the ejection efficiency of the ions from the source region. The 47+ ion exhibits a far lower ejection efficiency due to the reduced charge (unpublished, Schrader et al., manuscript in preparation). As a result of the low signal intensity (in the single ion detection regime), a slightly wider isolation window, a 61.12/38.88 duty cycle, was required. To plot the mass spectrum, an intensity cutoff was used to generate a list of *m*/*z* values for the detected ions. A histogram of these values was used to plot Figure 6f. Improvements in signal intensity will allow for a better resolving power for this isolation.

## Conclusions

Capacitive coupling was used for the addition of a DC offset to a digitally operated quadrupole mass filter. For duty cycles larger or smaller than 50%, a voltage offset is introduced into the waveform that results in a Mathieu *a* value less than 0. Additionally, the RF voltage was reduced slightly by the capacitor as shown by the slightly reduced isolation frequencies when the capacitors were present. The *a* value can be returned to 0 by adjusting the DC voltages applied to the rods. This offset was demonstrated empirically by experimentally plotting the Mathieu stability diagram. The ability to select single-charge states of protein complexes up to 800 kDa (13,000 *m*/*z*) is demonstrated for stability zone 1,1 with a resolution of approximately 280 at *m*/*z* 12,315. Low signal intensity for the charge-reduced GroEL complex reduced the attainable resolution at *m*/*z* 17,000.

The resolution achievable by the quadrupole is limited by the pseudopotential well depth, which can be increased by increasing the RF voltage, though this increases the fringing fields and increases the power requirements for the digital waveform generator. Future studies will focus on the isolation of protein complexes using higher stability zones beyond zone 1,1. These stability zones are advantageous because of their higher resolution and greater pseudopotential well depth for a given RF voltage.^38^ Accessing these zones will allow for higher-resolution isolation and further decrease the RF voltage requirements.

## Supporting Information Available

Instrument diagram, experimental Mathieu stability diagram plotted using alternative definition of U and V, and Mathieu stability diagrams of the apex with duty cycles near 61.2/38.8 (PDF)
