# Supplementary material for: Optimization of a Digital Mass Filter for the Isolation of Intact Protein Complexes in Stability Zone 1,1
Source: Anal Chem. 2023 Jan 26;95(5):3062–8. doi: 10.1021/acs.analchem.2c05221 (PMC9983038; doi:10.1021/acs.analchem.2c05221)
Supplement: Supplementary file 1 — ac2c05221_si_001.pdf [file ac2c05221_si_001.pdf]

Supplementary Materials

Optimization of a Digital Mass Filter for the Isolation Intact Protein Complexes in Stability Zone 1,1

Robert L. Schrader[1], Thomas E. Walker[1], Sumeet Chakravorty[2], Gordon A. Anderson[3], Peter T. A. Reilly[2], and David H. Russell[1,*]

[1]Department of Chemistry, Texas A&M University, College Station, TX 77843
[2]Department of Chemistry, Washington State University, Pullman, WA 99164
[3]GAA Custom Engineering, Kennewick, Washington 99338, United States

*Corresponding author; email: russell@chem.tamu.edu

Table of Contents

	Page
Figure S1	S2
Figure S2	S3
Figure S3	S4

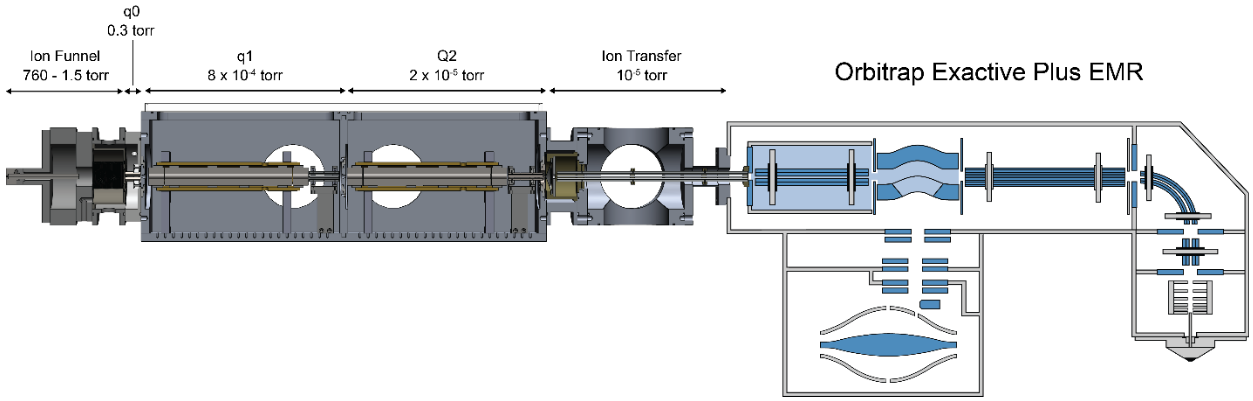

Figure S1. Solidworks rendering of the instrument interfaced with the rear of the HCD cell of the Orbitrap Exactive Plus EMR with major components labeled with approximate pressures of each vacuum region.

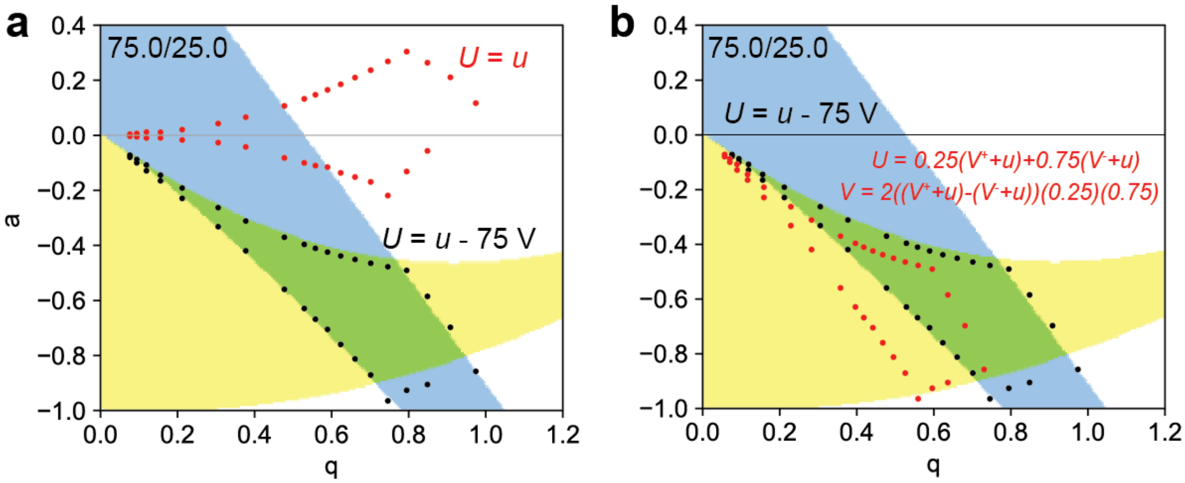

Figure S2. Mathieu stability diagram calculated from matrix methods for a 75.0/25.0 duty cycle superimposed with (a) experimentally calculated boundaries using standard *U* and *V* definitions and (b) using *U* and *V* values calculated from equations from Ding *et al.*, *Int. J. Mass Spectrom*. **2002**, *221*, 117-138. The experimental points calculated using these equations do not match the theoretical stability diagram as they do when using the standard *U* and *V* definitions. Note that the equation for *U* from Ding *et al.* simplifies to the equation used here for the RF voltage used here (150 V).

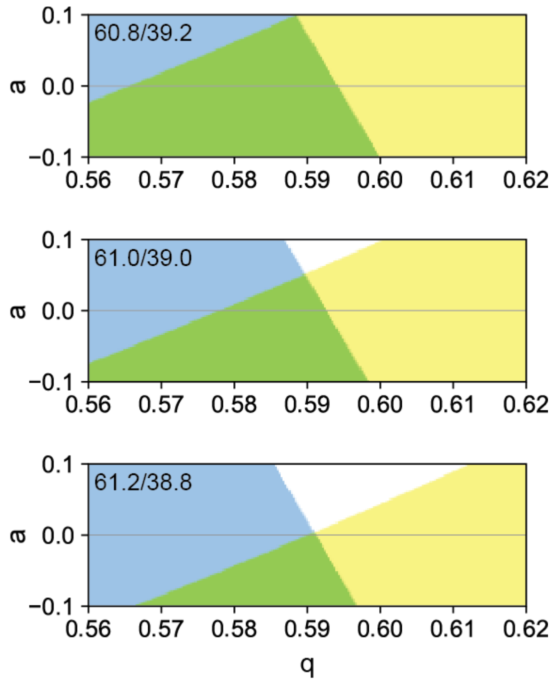

Figure S3. Stability diagrams for the apex of the stability diagram in Zone 1,1 for various duty cycles. The stability diagram is manipulated by the duty cycle and results in a tightening mass window at $a = 0$.
